# End-stage kidney disease in patient with epidermolysis bullosa - what are the treatment options? – case report

**DOI:** 10.1186/s12882-017-0606-6

**Published:** 2017-06-14

**Authors:** Michał Małecki, Maciej Domański, Kazimierz Ciechanowski

**Affiliations:** Department of Nephrology, Transplantation and Internal Medicine, Pomeranian Medical University, Al. Powstańców Wielkopolskich 72, 70-111 Szczecin, Poland

**Keywords:** Epidermolysis bullosa, Peritoneal dialysis, Haemodialysis, Kidney transplantation

## Abstract

**Background:**

Epidermolysis bullosa is a group of diseases caused by mutations in genes for proteins responsible for cells’ anchorage at the dermo-epidermal junction. Their common feature are dysfunctional or even absent connections between cells. The typical clinical sign is the formation of blisters, with possible excessive scarring, in response to minimal skin irritation. End stage renal disease may be one of the comorbidities in patients with epidermolysis bullosa. The implementation of renal replacement therapy may be very difficult in this population. This is mainly due to problems in obtaining the proper dialysis access. The choice of appropriate method may be crucial for patient’s survival.

**Case presentation:**

We present a case of 29-year-old woman with Epidermolysis bullosa caused by laminin 5 gene mutation. The patient suffered from additional conditions: blindness, complete bilateral sensorineural deafness and oesophageal stenosis. When end stage renal disease was diagnosed, the problem of renal replacement therapy had to be faced. There have been few reports concerning ESRD in this specific group of patients in the available literature. In most of them the prognosis was very poor. Nevertheless, we were very determined to overcome all the difficulties. Special procedures and cooperation with the patient’s family allowed us to consider kidney transplantation as a treatment option.

**Conclusion:**

There should be no limitations in renal replacement therapy in patients with epidermolysis bullosa. Haemodialysis, peritoneal dialysis and kidney transplantation are all possible treatment options. Nevertheless, either method requires special procedures.

## Background

Epidermolysis bullosa (EB) is a heterogeneous group of diseases inherited in an autosomal dominant or an autosomal recessive manner, characterized by fragility of the skin and mucous membranes with tendency to separate from underlying tissues. It leads to blisters and ulcers formation as a response to minor trauma. In some cases, extensive scar formation may be the result. The underlying causes are alterations in structure and function of proteins responsible for dermoepidermal junction integrity. Four major types of the disease have been described (further subdivided into 32 subtypes). Each type is characterized by a distinct pattern of tissue separation. In EB simplex (EBS, 92% of cases) skin separation occurs within the epidermis. In junctional EB (JEB, 1% of cases) it takes place at the level of lamina lucida, while in dystrophic EB (DEB, 5% of cases) splitting is located just beneath the lamina densa. Kindler syndrome, the fourth major type of EB, affects multiple layers of the skin. Some novel classifications are possible in the future as more mutations are diagnosed with the improvement of genetic diagnostics methods [1].

The disease affects 1 in 17,000 live births. The most common “simplex” type, is associated just with skin damage, while in other types internal organs are involved. The main morbidity and mortality causes in the population are squamous cell skin cancer and sepsis originating from infected skin lesions [1–3].

In most cases, the first clinical signs occur in early childhood or infancy. Nowadays available treatment options are focused on limiting the severity of the skin symptoms. The molecular or genetic therapies are still in preclinical trials [1, 4].

Kidney injury is observed mainly in the course of non-simplex EB type. It may be caused by ureter stenosis, glomerulonephritis or amyloidosis. It develops also as a result of systemic inflammatory response. Researchers from Great Ormond Street Hospital for Children in London observed different forms of urinary tract disorders like IgA nephropathy, post streptococcal glomerulonephritis, ureter obstruction, hypospodiasis in 5 juvenile patients during 10-year-long observation of dystrophic and junctional type. In one case the aetiology of kidney injury was unidentified [5]. In some of the EB types, end stage kidney disease is the common cause of death reaching even 12% as in patients with Hallopeau-Siemens (subtype of DEB). Nevertheless, as only minority of EB types involves internal organs, information on the kidney diseases in those patients are scarce. Unfortunately, the available reports indicate, that some patients died due to lack of treatment possibilities (no vascular access for haemodialysis) [6, 7]. On the other hand, some possibly lethal complications of the RRT may appear. The question arises therefore, which of the RRT methods is the most suitable for this specific group of patients.

## Case presentation

A 29-year-old female patient with Non Herlitz Type Epidermolysis Bullosa (one of the subtypes of JEB) has been under the care of dialysis unit of the University Hospital in Szczecin, Poland since 2011. The symptomatic disease was diagnosed early in childhood and confirmed genetically with the test for mutation in laminin 5 gene. Parents were diagnosed as mutation carriers. Additionally, the patient suffers from blindness of the right eye, bilateral complete sensorineural deafness and oesophageal stenosis. Her mental status has been slightly affected mainly by the limitations of social contacts, but she eventually graduated from high school.

At the age of 26 the ESRD was diagnosed during acute deterioration of kidney function. The patient was qualified for dialysis in emergency protocol, because of creatinine concentration 10 mg/dl (eGFR <10 ml/min/1,73 m2), severe metabolic acidosis and hyperkalaemia found at admission. The patient was introduced into haemodialysis program with acute catheter in the right internal carotid vein as vascular access. The cause of chronic kidney disease remained unknown. The history revealed recurrent urinary tract infections and active urine sediment in the past. The kidneys were small (8 cm in diameter) with increased echogenicity and decreased corticomedullary ratio. The previous documentation of abdominal ultrasound from 2007 provided no signs of chronic or acute kidney injury. Kidney function tests were within normal range then.

### Treatment modalities

After patient’s condition stabilized on haemodialysis, an attempt to start peritoneal dialysis was taken. The main reason for such decision was the danger of recurrent vascular access related infections. Unfortunately, shortly after Tenckhoff catheter insertion, the method had to be abandoned due to the massive peritoneal fibrosis and catheter occlusion, further complicated by dialysis peritonitis. Therefore, we decided to continue haemodialysis. Acute jugular dialysis catheter was replaced by tunnelled permanent catheter. We had to exchange the catheter twice during 2 years of haemodialysis due to catheter related infections with *Staphylococcus epidermidis* MRSE. Eventually catheter was placed in left jugular vein. Each of the procedures was complicated by slight damage to the epidermis with blister formation secondary to surgical manipulations. The irritation healed fast and with good effect, but required special dressing.

Considering the high risk of infectious complications related to dialysis catheter, it was decided to create an AV Fistula in the left forearm on 18th June 2014. Again, some worries concerning wound healing and blister formation following recurrent needle insertion appeared, but finally catheter-related infections encouraged us to qualify the patient for the aforementioned procedure. The surgery was complicated by local bleeding from the anastomosis, nevertheless there were no significant consequences and the scar healed properly. The adequate flow through fistula was achieved with reoperation. During fistula maturation, haemodialysis was continued according to standard plan using an additional dose of low molecular weight (every second day) to avoid thrombosis. Three months were necessary for the fistula maturation, which required an appropriate exercises performed by the patient. After the successful initiation of haemodialysis with new vascular access, the intravenous catheter was removed. The pain during the process of needle insertion was handled by local lignocaine ointment. Special dressings (silicone based Mepilex transfer) for patients with EB were used for covering and gentle pressure application to puncture sites. No tissue damage or severe blisters developed over fistula skin area. A qualification for kidney transplantation is in progress.

### Discussion

Kidney diseases in patients with EB are of different origin, in some cases kidney damage is post renal due to ureter obstruction (congenital or acquired), while in other cases it is believed that chronic inflammation causes glomerulonephritis due to immunological complexes formation or extensive immunoglobulin synthesis. In some cases, kidney failure is due to septic shock following infection from skin lesions [8, 5].

In our patient, chronic glomerulonephritis (active urinary sediment on time when end stage kidney failure was diagnosed) was the most probable reason for end stage renal disease. An ultrasound, performed on admission, showed small and fibrotic kidneys, therefore the biopsy was not performed.

There is a paucity of literature concerning patients with EB and ESRD. In some types of EB kidney failure occurrence may reach 12% - but this concerns most commonly EB subtypes which affect internal organs. It seems kidney failure is a casuistic phenomenon in patients with other types of EB, especially in EBS [8, 5].

It seems EB is not a condition, which could delay renal replacement therapy implementation, however we struggled for a longer time than usual with starting the dialysis in our patient due to lack of experience with such patients both in our country and in the world. The literature reveals some cases of patients who died of uraemia, as there was no possibility to establish a proper haemodialysis access [6]. There are only few more patients with the same type of EB in Poland – none of them required renal replacement therapy.

After obtaining some information about the disease, possibilities of surgical procedures and the risk of complications due to skin damage, we decided to start haemodialysis on intravenous catheter. The effects proved to be better than we expected. Not only patient’s condition improved, but we also noticed less skin lesions after few courses of haemodialysis. Intravenous catheter appears to be possible, but problematic option of the treatment. Our patient required few procedures of exchanging the catheter due to thrombosis or infection. Observing such situation, we focused on arteriovenous fistula creation. The available literature only reports the successful establishment of long-term dialysis access [9, 7]. In our patient, wound healing and occurrence of stenosis at the vessels junction point was the biggest concern, both for nephrologists and surgeons. Our optimism though was based on the literature review of few cases operated for other reasons [10, 11]. Nevertheless we were still facing the problem of recurrent needle insertion causing skin damage, which is considered an important problem in this group of patients. Our patient, her parents and our team have put a lot of effort to keep the fistula working. It required some patience, as the process of maturation was longer than in other patients.

Another problem, that may emerge is the pain following the procedure of needle insertion. After few attempts though, the pain became easily resistible (covering the skin with lignocaine gel was required before the procedure). The dialysis catheter was removed after first few successful uses of fistula – the benefit of avoiding recurrent catheter related infections is of significant importance. As far as we know, our patient is the only dialyzed patient suffering from EB to have arteriovenous fistula working for so many years.

There are some reports indicating a possibility of peritoneal dialysis in this population (mainly patients with DEB) [12, 13]. Probably good teamwork, patience and good communication between the patient, his family, renal team, surgeon and dermatologist are critical for success in EB patients. According to our experience peritoneal dialysis does not seem to be a good treatment option at least in patients with JEB. Excessive fibrosis that occurs around the Tenckhoff catheter makes it impossible to provide treatment with peritoneal dialysis (PD).

As mentioned earlier there have been many reports on surgical procedures in patients with EB [14, 15, 16–19]. Moreover, a report of successful kidney transplant in patient with the same type of EB appeared in March of 2014. The first successful attempt of kidney transplantation in patient with EB (Non Herlitz type – the same as our patient) was conducted in 2014 in Birmingham UK – the kidney function and patient’s condition has been stable during first year observations. The immunosuppressive protocol consisted of low dose tacrolimus, mycophenolate mofetil and prednisolon [20]. The Birmingham team’s success eventually encouraged us take another step toward renal replacement therapy in our patient. We started preparations for kidney transplantation. The biggest challenge at the moment appears to be the necessity of conducting the endoscopy. The difficulty presents itself in patient’s oesophageal stenosis. Other tests are not an obstruction to qualify the patient as kidney transplant recipient. Unfortunately, the same doubts resurface: the wound healing, probable stenosis in anastomosis area, the increased risk of developing SCC due to immunosuppressive therapy. All those factors make assessment of life expectancy while being dialyzed compared to receiving transplanted organ very hard. It becomes even more difficult when we realize how much has been achieved so far with good haemodialysis tolerance, proper vascular access, and lack of serious complications. Data on patients with EB attending surgery is promising. Lifesaving operations like appendectomy or even cardio surgical procedures are performed without any further complications [14, 15, 18, 19]. We expect however, that our patient is at higher risk of adhesions and fibrosis in the abdomen even though the operation itself does not involve opening the peritoneal cavity.

Hypothetical immunosuppression in this group might consist of antiproliferative drugs from m-TOR inhibitor group, like sirolimus or everolimus instead of calcineurin inhibitors to reduce the risk of skin cancer [21]. The main side effect of this group of drugs is wound healing delay but this could be turned beneficial for the patient, preventing fibrosis, adhesions, and stenosis in anastomosis regions. Cyclosporine A and mycophenolate mofetil have emerged lately as a possible treatment option in patients with dystrophic type of EB [22], therefore some improvement in skin lesions is also possible after induction of immunosuppressive therapy.

## Conclusions

Acute or chronic kidney failure may appear in patients with non-simplex type EB.

The vulnerability of epithelial tissues remains the gravest concern in qualification for surgery including dialysis catheter insertion or AV fistula creation in EB patients. Nevertheless, many surgical procedures were conducted without significant adverse effects.

Treatment of end stage kidney disease using haemodialysis is advised. Catheter insertion and fistula creation are both possible accesses for haemodialysis. Peritoneal dialysis is possible but objective difficulties as extensive fibrosis may appear.

Kidney transplantation in EB patients is possible – one successful case of such procedure is described in literature. The surgical complications and the choice of immunosuppressive therapy remains the main concern in the group of patients.

## Abbreviations

AV fistula: Arteriovenous fistula
DEB: Dystrophic epidermolysis bullosa
EB: Epidermolysis bullosa
EBS: Epidermolysis bullosa simplex
JEB: Junctional epidermolysis bullosa
PD: Peritoneal dialysis
RRT: Renal replacement therapy
SCC: Squamous cell carcinoma
